# Diarrheagenic Pathogens in Polymicrobial Infections

**DOI:** 10.3201/eid1704100939

**Published:** 2011-04

**Authors:** Brianna Lindsay, T. Ramamurthy, Sourav Sen Gupta, Yoshifumi Takeda, Krishnan Rajendran, G. Balakrish Nair, O. Colin Stine

**Affiliations:** Author affiliations: University of Maryland Baltimore, Baltimore, Maryland, USA (B. Lindsay, O.C. Stine);; National Institute for Cholera and Enteric Disease, Kolkata, India (T. Ramamurthy, S. Sen Gupta, Y. Takeda, K. Rajendran, G.B. Nair)

**Keywords:** Diarrhea, Vibrio infections, rotavirus, Vibrio cholerae, coinfection, bacteria, viruses, parasites, research

## Abstract

During systematic active surveillance of the causes of diarrhea in patients admitted to the Infectious Diseases and Beliaghata General Hospital in Kolkata, India, we looked for 26 known gastrointestinal pathogens in fecal samples from 2,748 patients. Samples from about one-third (29%) of the patients contained multiple pathogens. Polymicrobial infections frequently contained *Vibrio cholerae* O1 and rotavirus. When these agents were present, some co-infecting agents were found significantly less often (p = 10^–5^ to 10^–33^), some were detected significantly more often (p = 10^–5^ to 10^–26^), and others were detected equally as often as when *V. cholerae* O1 or rotavirus was absent. When data were stratified by patient age and season, many nonrandom associations remained statistically significant. The causes and effects of these nonrandom associations remain unknown.

The estimated worldwide death rate from diarrheal diseases is ≈2.2 million deaths per year (*1*). Diarrheal infections may be caused by an array of bacterial, viral, or parasitic pathogens. Some cases have 1 single defined cause, others do not have any defined cause, and a substantial number (one third) are caused by multiple pathogens (*2*). Because each known diarrheal pathogen fulfills Koch’s postulates and is capable of being the sole etiologic agent causing disease, multiple pathogens are not essential for causing disease. How additional pathogens cause and contribute to the disease process is unknown. The source of the multiple pathogens in a patient could simply result from multiple pathogens in an urban environment of crowded, impoverished conditions. If the various pathogens occurred independently in cases of disease, then each pathogen in a polymicrobial infection would be expected to occur in proportion to its presence in all patients with severe diarrhea.

In Kolkata, India, a megacity with a population >10 million, many persons live in crowded urban slums. Medical attention is available at the Infectious Disease and Beliaghata General Hospital, which serves the population of Kolkata. To determine the extent of disease caused by various bacterial, viral, and parasitic pathogens of the gastrointestinal tract, the National Institute of Cholera and Enteric Disease is conducting a systematic survey of patients hospitalized for diarrhea at this hospital. Analyses conducted after 2 years of data collection revealed that approximately one-third (29%) of patients had polymicrobial infections (*2*); an earlier report from that ongoing study indicated that the 3 parasites detected most often (in 73% of patients with polymicrobial infections) were *Giardia lamblia*, *Entamoeba histolytica*, and *Cryptosporidium* spp (*3*). We used data from the same ongoing survey to identify gastrointestinal tract pathogens in the feces of patients with severe diarrhea and to examine the relationships between co-infections of *Vibro cholerae* O1 and rotavirus with other bacterial, viral, and parasitic pathogens.

## Methods

Details of sample collection and microbiological analyses have been published (*2*). The protocol has been approved by the Institutional Review Board at the National Institute of Cholera and Enteric Disease. Briefly, fecal specimens were collected systematically from patients entering the hospital from November 2007 through February 2010. Of note, the previous study analyzed data through October 2009; however, the systematic sampling is still ongoing. The specimens were collected from every fifth patient with diarrhea on 2 randomly selected days each week. Only patients with diarrhea (defined by World Health Organization guidelines as passage of >3 loose or liquid stools per day or more frequently than is normal for the person) were eligible for inclusion in the study. Samples were collected from an average of 5.6% of eligible patients.

Each patient contributed 1 sample, and each sample was tested for all 26 common diarrheagenic pathogens. Standard microbiological techniques were used to examine the samples. Samples were collected in McCartney bottles (using sterile catheters or rectal swabs) containing Cary-Blair medium and examined (within 2 hours of collection) for bacterial, viral, and parasitic pathogens by a combination of conventional, immunologic, and molecular methods. The bacterial pathogens (*V. cholerae*, *V. parahaemolyticus*, *V. fluvialis*, *Campylobacter jejuni*, *Campylobacter coli*, *Salmonella* spp., *Shigella* spp., and diarrheagenic *Escherichia coli*) were isolated from appropriate selective media and identified by standard biochemical tests. Species and subtypes were confirmed by serotyping (for *V. parahaemolyticus, Shigella* spp., and *Salmonella* spp.) with commercially available antiserum (Denka Seiken, Tokyo, Japan; BioRad, Marnes-la-Coquette, France) and by PCR (for *V. cholerae* [*4*], *V. fluvialis* [*5*], enterotoxigenic *E. coli* [ETEC, including heat-labile and heat-stable enterotoxin producers], enteropathogenic *E. coli* [EPEC, typical and atypical], enteroaggregative *E. coli* [EAEC] [*6*], enteroinvasive *E. coli*, and Shiga toxin–producing *E. coli* [*7*]). Rotavirus was detected by polyacrylamide gel electrophoresis and silver staining (*8*). Noroviruses (groups I [NVG1] and II [NVG2]), sapovirus, and astrovirus were detected by reverse transcription–PCR with random primers for reverse transcription and specific primers for PCR (*9*). Adenoviruses were detected by the commercially available RotaAdeno VIKIA Kit (bioMérieux, Marcy l’Etoile, France). All samples were screened by using a highly sensitive antigen capture ELISA (TechLab, Inc., Blacksburg, VA, USA) of *G. lamblia*, *Cryptosporidium parvum, E. histolytica*, and *Blastocystis hominis.*

To test for possible associations, we used the Fisher exact test to compare pairs of pathogens (1, both, or neither) with an independent assortment based on the overall frequency with which pathogens were detected. To establish criteria for statistical significance, we calculated p values, odds ratios (ORs), and 95% confidence intervals (CIs). Additional covariates were collected and examined for confounding and interaction. These included patient age, gender, residence, and religion and season of infection. Seasons were defined as summer (March–June), monsoon (July–October), and winter (November–February). All analyses were conducted by using SAS version 9.2 (SAS Institute, Cary, NC, USA).

## Results

Fecal samples were submitted from 2,748 patients. Patient demographic characteristics are listed in Table 1. A large proportion (44%) of patients were 15–45 years of age, ≈13% were <1 year of age, 80% resided in urban areas, 74% were Hindu, and 25% were Muslim. The following pathogens were detected in at least 1 sample: adenovirus, *Aeromonas* spp., astrovirus, *B. hominis*, *C. jejuni*, *C. parvum,* EAEC, EPEC, ETEC, *E. histolytica*, *G. lamblia*, NVG1, NVG2, rotavirus, *Salmonella* spp., sapovirus, *Shigella* spp., *V. cholerae* O1, *V. cholerae* O139, *V. cholerae* non-O1, *V. cholerae* non-O139, *V. parahaemolyticus*, and *V. fluvialis*. No pathogens were detected in 766 (28%) of the 2,748 samples (Table 1), but test results were positive for the other 72%. One pathogen was found for 1,169 (43%) samples and multiple pathogens for 813 (29%) (Table 1). The 2 most commonly detected pathogens were *V. cholerae* O1 and rotavirus, which were found in 24% and 22% of samples, respectively.

**Table 1:** Characteristics of 2,748 patients hospitalized with diarrhea, Kolkata, India, November 2007–February 2010*

Characteristic	Total, no. (%)
No. pathogens	
0	766 (27.9)
>1	1,982 (72.1)
1	1,169 (42.5)
2	589 (21.4)
3	165 (6.0)
4	44 (1.6)
5	10 (0.4)
6	5 (0.2)
Age group, y*	
<1	360 (13.1)
>1–2	233 (8.5)
>2–5	177 (6.4)
>5 –15	243 (8.8)
>15 –45	1,210 (44.0)
>45	525 (19.1)
Gender	
M	1,482 (53.9)
F	1,266 (46.1)
Residence	
Urban	2,226 (81.0)
Rural	522 (19.0)
Religion	
Hindu	2,043 (74.3)
Muslim	698 (25.4)
Christian	5 (0.2)
Other	2 (0.1)
Season	
Nov–Feb	890 (32.4)
Mar–Jun	837 (30.5)
Jul–Oct	1,021 (37.1)
Feces	
Watery	2,080 (75.7)
Loose	561 (20.4)
Bloody	21 (0.8)
Mucoid	15 (0.5)
Bloody and mucoid	71 (92.6)

*Mean ± SD patient age 26 ± 22 y.

*V. cholerae* O1 was detected in 661 samples. *V. cholerae* was the sole pathogen in 379 samples; however, it was isolated along with another diarrheagenic pathogen from 282 samples. The co-infection of *V. cholerae* and rotavirus was highly significant (p = 1.12 × 10^–33^). Co-infection with *V. cholerae* and rotavirus was ≈5-fold less likely (OR 0.18, 95% CI 0.13–0.25; Figure, panel A) to occur among those with than among those without *V. cholerae* infection. A negative association might be expected if a case of severe diarrhea caused by any given pathogen excluded other pathogens. Consistent with this expectation, the presence of *C. parvum*, adenovirus, *Shigella* spp., ETEC, and *V. parahaemolyticus* was decreased significantly (p = 7.87 × 10^–5^ to 1.32 × 10^–9^) and was 12.5-fold (with *V. parahaemolyticus,* OR 0.1, 95% CI 0.02–0.33) to 2.44-fold (with *C. parvum*) less likely to occur among those with than among those without *V. cholerae* infection. However, antithetically, the rate of *G. lamblia* co-infection was significantly higher among *V. cholerae* O1–positive than among *V. cholerae* O1–negative fecal samples (OR 1.71, 95% CI 1.32–2.21). A significant difference in infection rates among those with and without *V. cholerae* O1 infection was not found for EAEC, *C. jejuni*, *V. fluvialis*, *E. histolytica*, astrovirus, NVGII, and EPEC. Tests for association were not performed for *Salmonella* spp., NVGI, *Aeromonas* spp., *B. hominis*, *C. coli*, sapovirus, *V. cholerae* non-O1, *V. cholerae* non-O139, and *V. cholerae* O139 because the low number of patients infected with those pathogens resulted in insufficient power.

**Figure:**
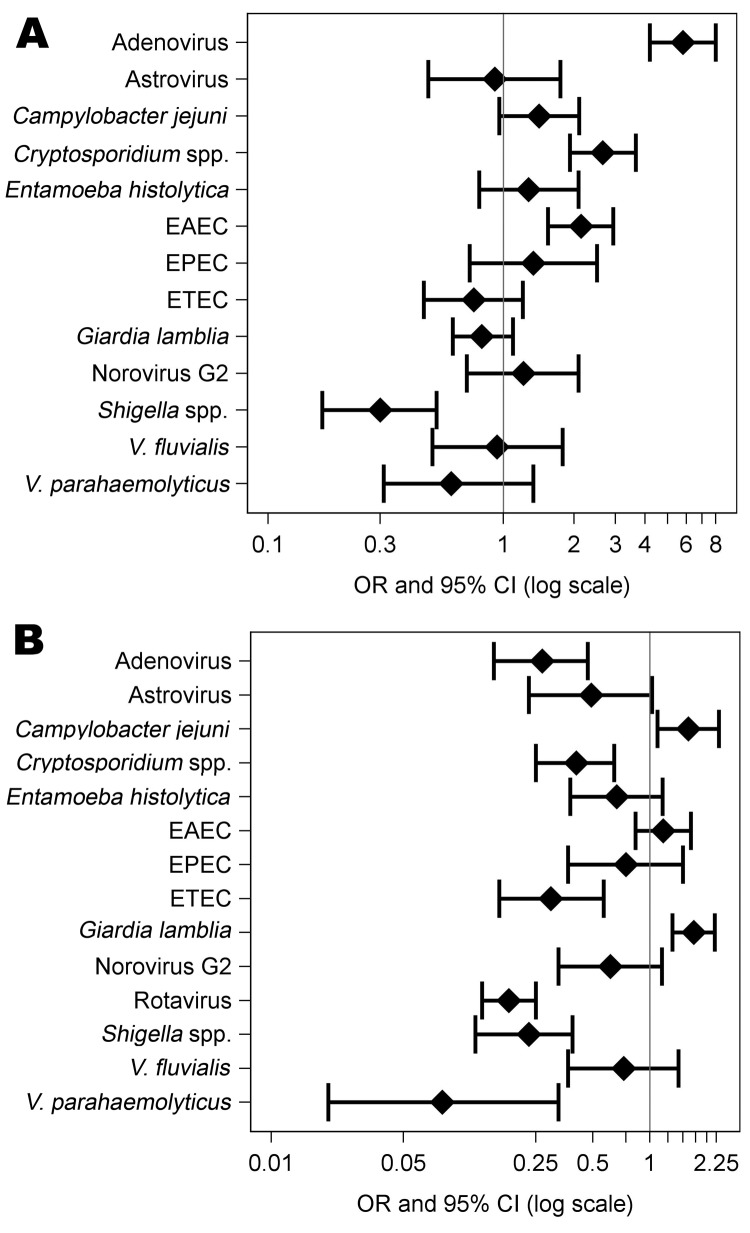
Odds ratios (ORs) showing odds of A) *Vibrio cholerae* or B) rotavirus co-occurring with various other pathogens relative to the odds of *V. cholerae* or rotavirus co-occurring independently with various other pathogens at the frequency with which each is present in the entire sample. This standard forest plot indicates the best estimate and the 95% confidence intervals (CIs) for each co-occurring organism. EAEC, enteroaggregative *Escherichia coli*; EPEC, enteropathogenic *E. coli;* ETEC, enterotoxigenic *E. coli.*

Rotavirus was detected in 594 of the fecal samples and was the sole pathogen found in 253 of them. Rotavirus and at least 1 other gastrointestinal pathogen were found in 341 samples; 119 samples were co-infected with rotavirus and >2 other pathogens. When the effect of rotavirus co-infection with other pathogens was tested (Figure, panel B), *Shigella* spp. were significantly less likely to be found in samples with rotavirus than in samples without rotavirus (OR 0.30, 95% CI 0.17–0.52). In contrast, EAEC, *Cryptosporidium* spp., and adenovirus were significantly increased in samples with rotavirus (p = 6.15 × 10^–6^ to 1.61 × 10^–26^; ORs 2.14–5.80. A significant effect was not observed for *G. lamblia*, *C. jejuni*, EPEC, ETEC, *V. parahaemolyticus*, *V. fluvialis*, *E. haemolyticus*, astrovirus, and NVGII. Tests for association were not performed for *Salmonella* spp., NVG1, *Aeromonas* spp., *B. hominis*, *C. coli*, sapovirus, *V. cholerae* non-O1, *V. cholerae* non-O139, and *V. cholerae* O139 because the low number of patients infected with those pathogens resulted in insufficient power. Analysis of samples from patients infected simultaneously with *G. lamblia*, *V. cholerae*, and rotavirus (n = 41) revealed that the frequency of co-infection with *G. lamblia* was not significantly affected by co-infection with *V. cholerae* O1 and rotavirus (p = 0.08).

Analysis of covariates indicated that gender, religion, and residence largely had no effect on the associations between pathogens; however, in some instances, age and season were identified as confounders or effect modifiers (Table 2). To examine the effect of these covariates, we stratified the data by age and season and found that many associations remained significant (Table A1). Associations between rotavirus and adenovirus remained significant for all age and season strata except among children <1 year of age. After adjusting for age and season by using logistic regression models, we found that co-infection with rotavirus and *Cryptosporidium* spp. and co-infection with rotavirus and *Shigella* spp. remained significant (Table 2). The negative association between *V. cholerae* and adenovirus remained significant after adjustment by logistic regression for age and season (OR 0.36; 95% CI 0.21–0.64); associations between *V. cholerae* and many other pathogens remained significant within specific strata of age and season. Assessing the effect of covariates was limited in some instances because of small cell sizes. For this reason, we did not include stratified results for co-infection with *V. cholerae* and *V. parahaemolyticus* in the Table A1.

**Table 2:** Effect of covariates on gastrointestinal pathogen associations*

Pathogens	Covariate					
	Age	Season	Gender	Residence	Religion	After adjusting for effects
*Vibrio cholerae*/rotavirus	Confounder	Interaction	No effect	No effect	No effect	Significant except for age strata 5–15 y
*V. cholerae*/adenovirus	Confounder	No effect	No effect	No effect	No effect	Significant when regression adjusted for age and season (OR 0.36, 95% CI 0.21–0.64)
*V. cholerae*/*Cryptosporidium* spp.	Confounder	Confounder	Interaction	No effect	No effect	Significant for female, not male patients; small stratified cell sizes
*V. cholerae*/*Giardia lamblia*	Interaction	Confounder	No effect	Interaction	No effect	Significant for some age categories
*V. cholerae*/*Shigella* spp.	Interaction	No effect	No effect	No effect	No effect	Significant for ages >2 y and all seasons
*V. cholerae*/ETEC	No effect	Interaction	No effect	No effect	No effect	Significant for summer and monsoon seasons
Rotavirus/adenovirus	Interaction	Interaction	No effect	No effect	No effect	Significant for all seasons and all age strata except <1 y
Rotavirus/*Cryptosporidium* spp.	Confounder	Confounder	No effect	No effect	No effect	Significant when regression adjusted for age and season (OR 1.64, 95% CI 1.11–2.41)
Rotavirus/EAEC	Confounder	No effect	No effect	No effect	Interaction	Not significant when regression adjusted for age and season (OR 1.38, 95% CI 0.94–2.01); significant for age strata 2–5 y; all seasons significant
Rotavirus/*Shigella* spp.	Confounder	Confounder	No effect	No effect	No effect	Significant when regression adjusted for age and season (OR 0.24, 95% CI 0.14–0.44)

*Interaction identified using Breslow-Day test for homogeneity, p<0.05. Considered statistically significant at p<0.05. OR, odds ratio; CI, confidence interval; ETEC, enterotoxigenic *Escherichia coli*; EAEC, enteroaggregative *E. coli*.

## Discussion

Our analyses revealed that co-occurrence of gastrointestinal pathogens in feces of patients with polymicrobial infections and severe diarrhea necessitating hospitalization was not in proportion to the pathogens’ presence in all patients with diarrhea. Tests for association were performed with *V. cholerae* O1 and rotavirus because they were the most commonly detected pathogens and, hence, had the greatest power to detect an association with the other pathogens. Some combinations of pathogens occurred less frequently than expected (e.g., *V. cholerae* and rotavirus [OR 0.18, 95% CI 0.13–0.25]); some combinations appeared more frequently than expected (e.g., rotavirus and adenovirus [OR = 5.8, 95% CI 4.20–7.99]), and some combinations occurred at the same frequency whether with or without *V. cholerae* or rotavirus. After adjustment for age and season, these variables often acted as confounders or effect modifiers, but in general the associations remained significant. However, many of the stratified analyses had small numbers for comparison.

*V. cholerae* O1 exhibited a positive association with only *G. lamblia*, suggesting that something may be unique about the co-occurrence of those 2 gastrointestinal pathogens. In support of that idea are 1) a report that co-infection with *G. lamblia* and *V. cholerae* results in *G. lamblia* being present in trophozoite form rather than in the cyst form found in feces of control patients (*10*), and 2) a previous finding that *G. lamblia* trophozoites can bind cholera toxin (*11*). Alternatively, each is a pathogen with substantial environmental reservoirs, and the positive association may simply represent acquisition of both pathogens from the same environmental source.

Phylogenetic relatedness alone does not explain the apparent competitive inhibition or negative association that we found between *V. cholerae* and other pathogens. For example, although the closely related *V. cholerae* O1 and *V. parahaemolyticus* exhibited a 10-fold negative association, *V. fluvialis*, which is phylogenetically only slightly farther from *V. cholerae* O1 than is *V. parahaemolyticus*, did not show any inhibition in the presence of *V. cholerae*. Also, although 2 members of the family *Enterobacteriaceae* (*Shigella* spp. and ETEC) were found less frequently than expected in combination with *V. cholerae* O1, 2 other members of that family (EPEC and EAEC) occurred in the expected proportion in samples from patients with mixed *V. cholerae* O1 infections.

Rotavirus had multiple strong positive associations (ORs >2) with the other gastrointestinal pathogens detected in the fecal samples, even after considering age and season. The positive association of rotavirus, an RNA virus that affects cells covered in microvilli, and adenovirus, a DNA virus that affects cells that are dividing to generate new cells with microvilli, may represent an interaction between pathogens to cause more severe diarrhea (in our sample all patients were hospitalized) in patients 15–45 years of age (OR 10.81, 95% CI 5.63–20.78) or a way to escape or circumvent immunity from previous exposures. In a previous study, Koh et al. (*12*) found that among children in Korea with virus-caused diarrhea, adenoviruses occurred preferentially in the presence of rotavirus and that rotavirus and norovirus, although most common, occurred in polymicrobial infections in proportion to their numbers in the samples. Both observations are consistent with the results of our study. Furthermore, Bilenko et al. (*13*) observed that among Bedouins, *G. lamblia* was frequently found in polymicrobial infections and, when present with rotavirus, produced less severe diarrhea than rotavirus alone. In addition, Souza et al. (*14*) found that among young children in São Paulo, those with rotaviral and bacterial co-infections were more likely to have severe diarrhea than were children infected with either pathogen alone. However, the study had insufficient power to examine differences among bacterial pathogens, as did the rest of the studies of rotavirus infections mentioned in a recent review (*15*).

The presence of multiple pathogens in one third of patients with diarrhea has potential implications for treatment and raises several questions. Do cases of diarrhea caused by *V. cholerae* or rotavirus and a second pathogen differ from those caused by *V. cholerae* or rotavirus alone? Does 1 pathogen lead the way for another to successfully infect a person? Do the pathogens behave synergistically to escape immunologic detection? Because the cross-sectional nature of our study did not enable us to investigate the temporal sequence of pathogen infection, future research is needed to provide more evidence concerning the causal pathway(s). Also, the clinical significance of our findings must be more rigorously evaluated by studies that include infected patients and controls. A more substantive investigation into how age and season might affect polymicrobial infections should also be conducted.

The results of our current study indicate that associations can occur between some pathogens affecting the human gastrointestinal tract. The observation of selective positive associations among some gastrointestinal pathogens raises the question of how they interact in vivo; e.g., is the critical factor a modification of gastrointestinal tract microflora? Understanding the association(s) among various co-infecting pathogens may help direct the development of treatment strategies.

## Appendix

**Table A1:** Stratified odds ratios comparing the odds of *Vibrio cholerae* or rotavirus co-occurring with various other pathogens, relative to the odds of *V. cholerae* or rotavirus co-occurring independently with various additional pathogens at the frequency with which each is present in the entire sample*

*Vibrio cholerae*																			
Data	*Giardia lamblia*				*Shigella* spp.				ETEC				Adenovirus				*Cryptosporidium* spp.		
	O	E	OR (95% CI)		O	E	OR (95% CI)		O	E	OR (95% CI)		O	E	OR (95% CI)		O	E	OR (95% CI)
Unadjusted OR	101	72	**1.71 (1.32–2.13)**		12	42	**0.22 (0.12–0.40)**		11	29	**0.3 (0.16–0.57)**		14	41	**0.27 (0.15–0.47)**		20	40	**0.41 (0.25–0.66)**
Age group, y																			
<1	8	3	**4.25 (1.72–10.5)**		1	1	1.10 (0.13–8.92)		0	1	†		2	5	0.35 (0.08–1.50)		3	4	0.68 (0.20–2.35)
>1–2	9	5	**2.77 (1.15–6.66)**		4	3	1.35 (0.43–4.24)		2	1	1.92 (0.38–9.96)		1	4	0.19 (0.02–1.45)		1	3	0.30 (0.04–2.34)
>2– 5	12	9	1.73 (0.76–3.91)		2	7	**0.18 (0.04–0.80)**		0	1	†		2	4	0.38 (0.08–1.77)		1	2	0.35 (0.04–3.04)
>5–15	17	18	0.82 (0.45–1.57)		2	7	**0.20 (0.04–0.90)**		1	3	0.24 (0.03–2.00)		3	4	0.74 (0.18–2.96)		2	3	0.57 (0.11–2.92)
>15–45	45	30	**1.95 (1.31–2.90)**		3	15	**0.14 (0.04–0.46)**		4	13	**0.22 (0.08–0.63)**		3	11	**0.21 (0.07–0.72)**		10	16	0.53 (0.27–1.05)
>45	10	9	1.09 (0.51–2.33)		0	10	**0.89 (0.86–0.93)**		4	9	**0.34 (0.12–0.99)**		3	4	0.59 (0.16–2.11)		3	6	0.43 (0.12–1.49)
Season																			
Winter	22	12	**2.36 (1.39–4.01)**		1	9	**0.09 (0.01–0.63)**		4	4	0.94 (0.32–2.75)		2	10	**0.17 (0.04–0.69)**		4	14	**0.24 (0.08–0.65)**
Summer	36	27	**1.62 (1.05–2.51)**		4	16	**0.18 (0.06–0.50)**		1	12	**0.06 (0.01–0.41)**		8	15	**0.43 (0.20–0.93)**		3	9	**0.27 (0.08–0.91)**
Monsoon	43	35	1.41 (0.94–2.12)		7	15	**0.36 (0.16–0.81)**		6	14	**0.34 (0.14–0.82)**		4	14	**0.21 (0.07–0.60)**		13	13	0.97 (0.50–1.88)
Rotavirus																			
Data	*Vibrio cholerae*				Adenovirus				*Cryptosporidium spp.*				EAEC				*Shigella spp.*		
	O	E	OR (95% CI)		O	E	OR (95% CI)		O	E	OR (95% CI)		O	E	OR (95% CI)		O	E	OR (95% CI)
Unadjusted OR	41	143	**0.18 (0.13–0.25)**		98	36	**5.79 (4.20–7.98)**		68	36	**2.65 (1.92–3.66)**		65	39	**2.14 (1.55–2.93)**		14	38	**0.30 (0.17–0.52)**
Age group, y																			
<1	6	18	**0.14 (0.05–0.35)**		38	35	1.30 (0.72–2.35)		32	29	1.28 (0.68–2.40)		33	30	1.42 (0.75–2.69)		4	7	0.36 (0.10–1.26)
>1–2	10	17	**0.35 (0.15–0.78)**		22	17	**2.30 (1.01–5.24)**		14	11	1.78 (0.69–4.60)		15	15	0.98 (0.44–2.15)		4	13	**0.14 (0.05–0.43)**
>2–5	6	15	**0.23 (0.09–0.58)**		7	3	**3.50 (1.11–11.01)**		2	2	1.07 (0.20–5.75)		9	4	**4.00 (1.40–11.5)**		0	6	†
>5–15	5	9	0.38 (0.14–1.05)		4	1	**6.40 (1.67–24.39)**		1	1	1.20 (0.14–10.15)		1	1	0.62 (0.07–5.00)		0	2	†
>15–45	6	31	**0.13 (0.06–0.30)**		20	4	**10.81 (5.63–20.78)**		12	6	**2.38 (1.23–4.62)**		5	5	0.91 (0.35–2.33)		3	6	0.48 (0.15–1.58)
>45	8	15	**0.41 (0.19–0.91)**		7	2	**6.27 (2.28–17.19)**		7	2	**4.13 (1.61–10.62)**		2	2	0.89 (0.20–3.94)		3	4	0.63 (0.19–2.12)
Season																			
Winter	5	47	**0.06 (0.02–0.13)**		42	23	**3.73 (2.20–6.33)**		45	32	**1.94 (1.25–3.00)**		29	21	**1.8 (1.06–3.04)**		6	21	**0.19 (0.08–0.44)**
Summer	21	37	**0.44 (0.27–0.72)**		30	10	**5.75 (3.34–9.91)**		11	6	**2.39 1.14–5.01)**		14	8	**2.28 (1.18–4.40)**		5	11	**0.38 (0.15–0.97)**
Monsoon	15	42	**0.24 (0.14–0.42)**		26	6	**11.48 (6.04–21.44)**		12	6	**2.66 (1.33–5.33)**		22	10	**2.91 (1.71–4.95)**		3	7	0.41 (0.13–1.34)

*ETEC, enterotoxigenic *Escherichia coli*, O, observed number of cases positive for both pathogens; E, expected number of cases positive for both pathogens; OR, odds ratio; CI, confidence interval; EPEC, enteropathogenic *E. coli*; EAEC, enteroaggregative *E. coli*. **Boldface** indicates statistical significance at p<0.05. †Cell counts too small to calculate OR.
